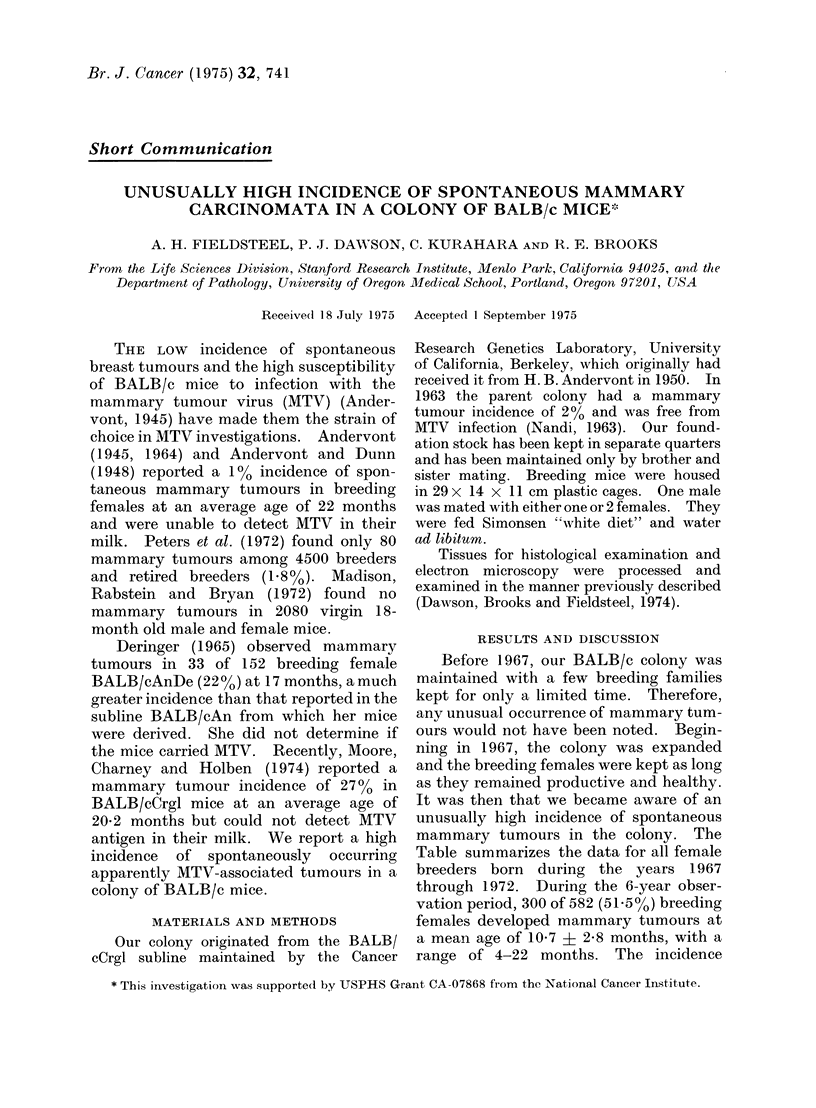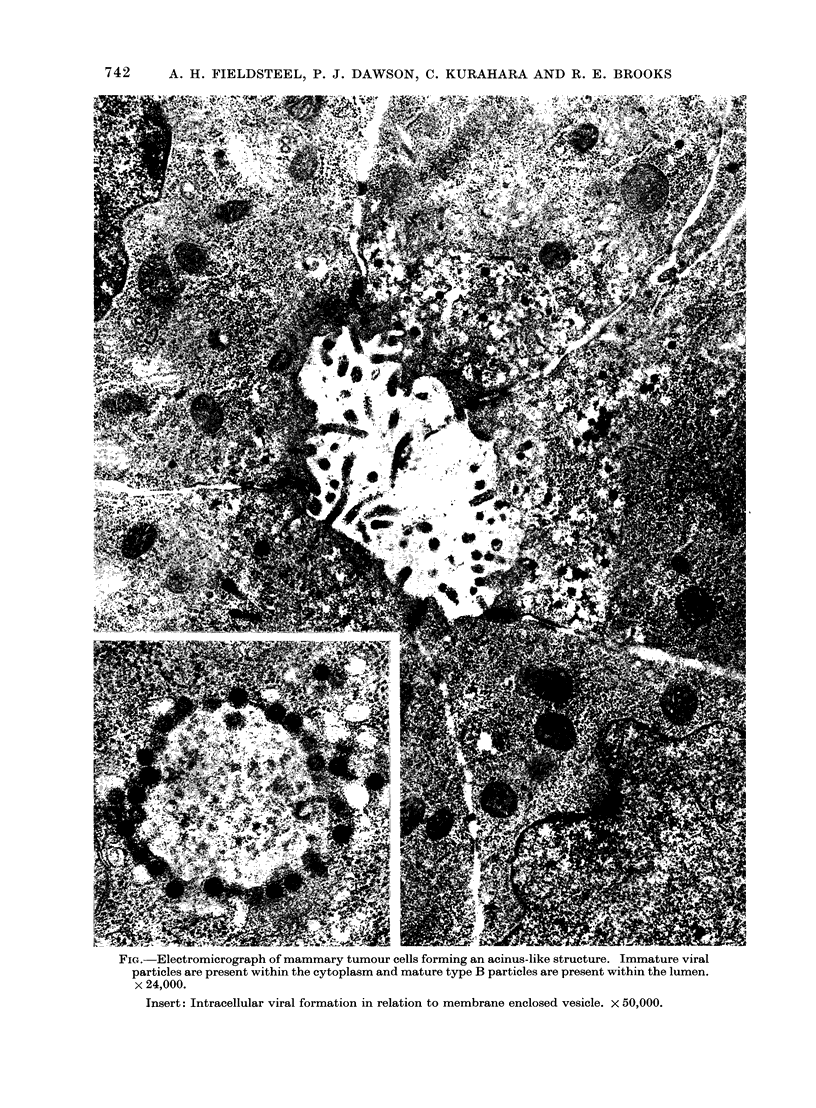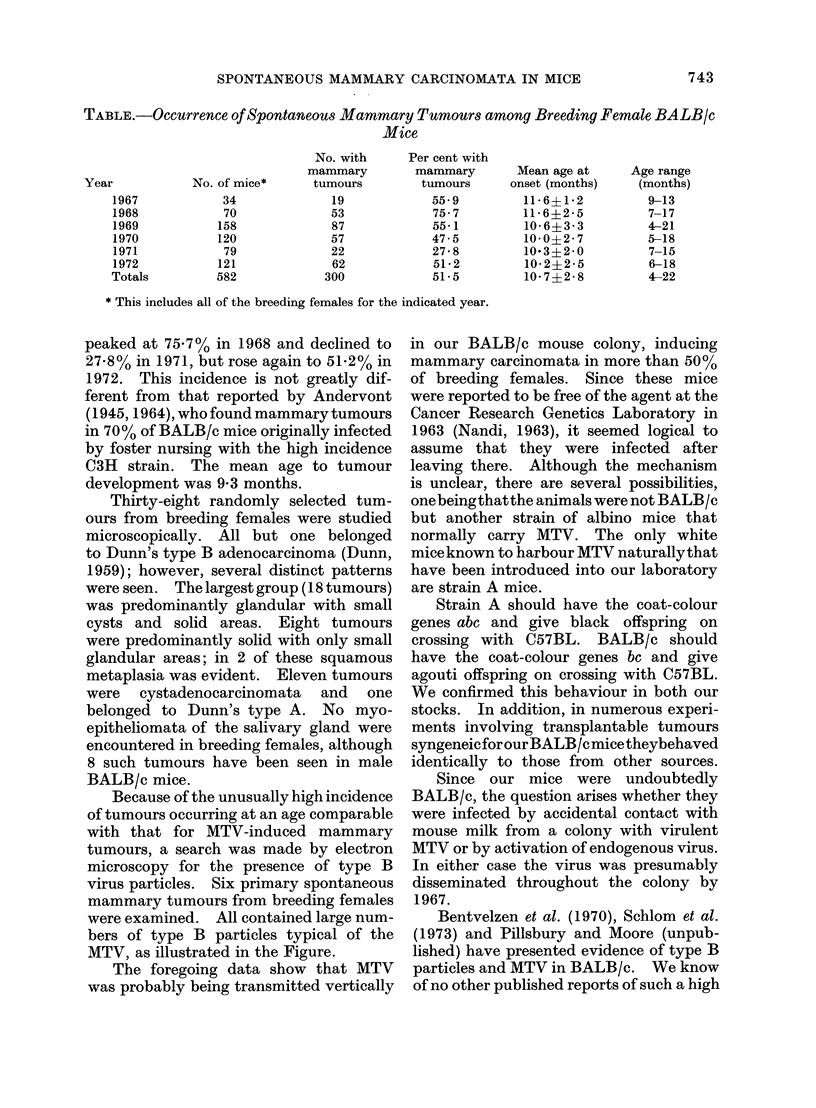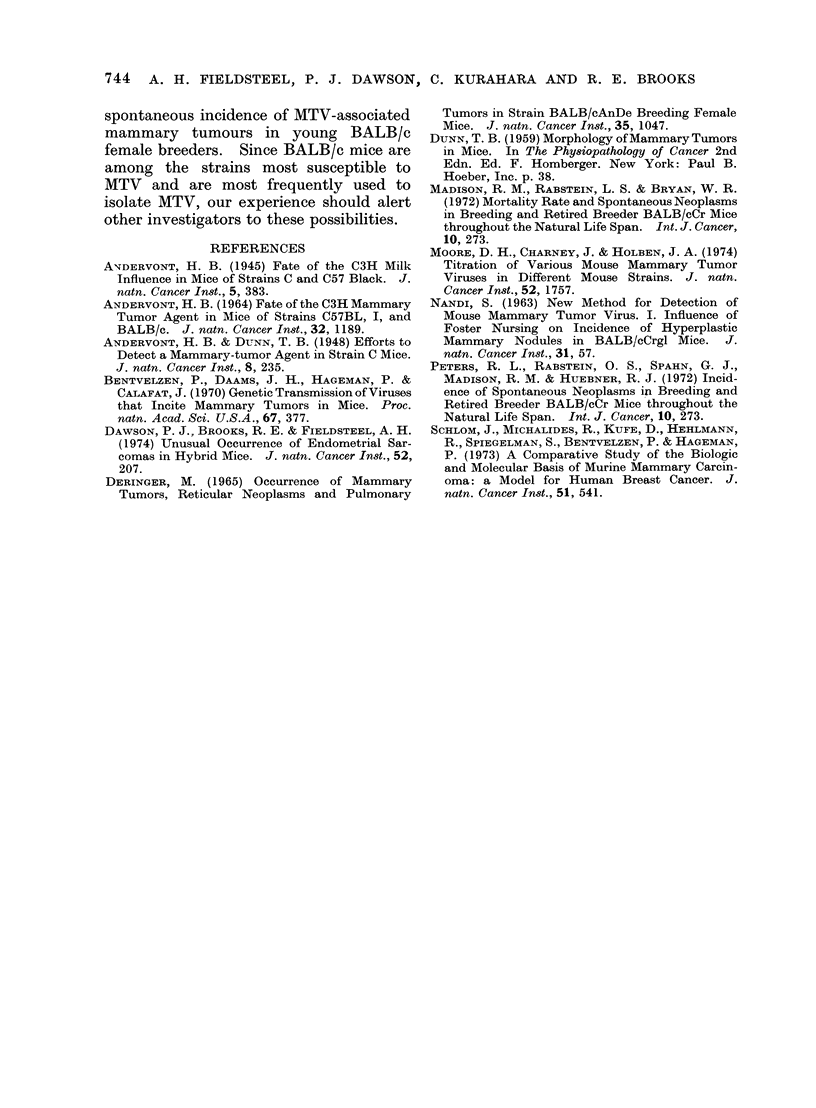# Unusually high incidence of spontaneous mammary carcinomata in a colony of BALB/c mice.

**DOI:** 10.1038/bjc.1975.286

**Published:** 1975-12

**Authors:** A. H. Fieldsteel, P. J. Dawson, C. Kurahara, R. E. Brooks

## Abstract

**Images:**


*Br. J. Cancer* (1975) **32**, 741

***Short Communication***

### UNUSUALLY HIGH INCIDENCE OF SPONTANEOUS MAMMARY CARCINOMATA IN A COLONY OF BALB/c MICE*

A. H. FIELDSTEEL, P. J. DAWSON, C. KURAHARA AND R. E. BROOKS

*From the Life Sciences Division, Stanford Research Institute, Menlo Park, California 94025, and the Department of Pathology, University of Oregon Medical School, Portland, Oregon 97201, USA*

Received 18 July 1975 Accepted 1 September 1975

THE LOW incidence of spontaneous breast tumours and the high susceptibility of BALB/c mice to infection with the mammary tumour virus (MTV) (Andervont, 1945) have made them the strain of choice in MTV investigations. Andervont (1945, 1964) and Andervont and Dunn (1948) reported a 1% incidence of spontaneous mammary tumours in breeding females at an average age of 22 months and were unable to detect MTV in their milk. Peters *et al.* (1972) found only 80 mammary tumours among 4500 breeders and retired breeders (1·8%). Madison, Rabstein and Bryan (1972) found no mammary tumours in 2080 virgin 18-month old male and female mice.

Deringer (1965) observed mammary tumours in 33 of 152 breeding female BALB/cAnDe (22%) at 17 months, a much greater incidence than that reported in the subline BALB/cAn from which her mice were derived. She did not determine if the mice carried MTV. Recently, Moore, Charney and Holben (1974) reported a mammary tumour incidence of 27% in BALB/cCrgl mice at an average age of 20·2 months but could not detect MTV antigen in their milk. We report a high incidence of spontaneously occurring apparently MTV-associated tumours in a colony of BALB/c mice.

### MATERIALS AND METHODS

Our colony originated from the BALB/cCrgl subline maintained by the Cancer Research Genetics Laboratory, University of California, Berkeley, which originally had received it from H. B. Andervont in 1950. In 1963 the parent colony had a mammary tumour incidence of 2% and was free from MTV infection (Nandi, 1963). Our foundation stock has been kept in separate quarters and has been maintained only by brother and sister mating. Breeding mice were housed in 29 × 14 × 11 cm plastic cages. One male was mated with either one or 2 females. They were fed Simonsen "white diet" and water *ad libitum.*

Tissues for histological examination and electron microscopy were processed and examined in the manner previously described (Dawson, Brooks and Fieldsteel, 1974).

### RESULTS AND DISCUSSION

Before 1967, our BALB/c colony was maintained with a few breeding families kept for only a limited time. Therefore, any unusual occurrence of mammary tumours would not have been noted. Beginning in 1967, the colony was expanded and the breeding females were kept as long as they remained productive and healthy. It was then that we became aware of an unusually high incidence of spontaneous mammary tumours in the colony. The Table summarizes the data for all female breeders born during the years 1967 through 1972. During the 6-year observation period, 300 of 582 (51·5%) breeding females developed mammary tumours at a mean age of 10·7 ± 2·8 months, with a range of 4–22 months. The incidence

* This investigation was supported by USPHS Grant CA-07868 from the National Cancer Institute.

FIG.—Electromicrograph of mammary tumour cells forming an acinus-like structure. Immature viral particles are present within the cytoplasm and mature type B particles are present within the lumen. $\times$ 24,000.

Insert: Intracellular viral formation in relation to membrane enclosed vesicle. $\times$ 50,000.

TABLE.—*Occurrence of Spontaneous Mammary Tumours among Breeding Female BALB/c Mice*

| Year | No. of mice* | No. with mammary tumours | Per cent with mammary tumours | Mean age at onset (months) | Age range (months) |
|---|---|---|---|---|---|
| 1967 | 34 | 19 | 55·9 | 11·6±1·2 | 9–13 |
| 1968 | 70 | 53 | 75·7 | 11·6±2·5 | 7–17 |
| 1969 | 158 | 87 | 55·1 | 10·6±3·3 | 4–21 |
| 1970 | 120 | 57 | 47·5 | 10·0±2·7 | 5–18 |
| 1971 | 79 | 22 | 27·8 | 10·3±2·0 | 7–15 |
| 1972 | 121 | 62 | 51·2 | 10·2±2·5 | 6–18 |
| Totals | 582 | 300 | 51·5 | 10·7±2·8 | 4–22 |

* This includes all of the breeding females for the indicated year.

peaked at 75·7% in 1968 and declined to 27·8% in 1971, but rose again to 51·2% in 1972. This incidence is not greatly different from that reported by Andervont (1945, 1964), who found mammary tumours in 70% of BALB/c mice originally infected by foster nursing with the high incidence C3H strain. The mean age to tumour development was 9·3 months.

Thirty-eight randomly selected tumours from breeding females were studied microscopically. All but one belonged to Dunn's type B adenocarcinoma (Dunn, 1959); however, several distinct patterns were seen. The largest group (18 tumours) was predominantly glandular with small cysts and solid areas. Eight tumours were predominantly solid with only small glandular areas; in 2 of these squamous metaplasia was evident. Eleven tumours were cystadenocarcinomata and one belonged to Dunn's type A. No myoepitheliomata of the salivary gland were encountered in breeding females, although 8 such tumours have been seen in male BALB/c mice.

Because of the unusually high incidence of tumours occurring at an age comparable with that for MTV-induced mammary tumours, a search was made by electron microscopy for the presence of type B virus particles. Six primary spontaneous mammary tumours from breeding females were examined. All contained large numbers of type B particles typical of the MTV, as illustrated in the Figure.

The foregoing data show that MTV was probably being transmitted vertically in our BALB/c mouse colony, inducing mammary carcinomata in more than 50% of breeding females. Since these mice were reported to be free of the agent at the Cancer Research Genetics Laboratory in 1963 (Nandi, 1963), it seemed logical to assume that they were infected after leaving there. Although the mechanism is unclear, there are several possibilities, one being that the animals were not BALB/c but another strain of albino mice that normally carry MTV. The only white mice known to harbour MTV naturally that have been introduced into our laboratory are strain A mice.

Strain A should have the coat-colour genes *abc* and give black offspring on crossing with C57BL. BALB/c should have the coat-colour genes *bc* and give agouti offspring on crossing with C57BL. We confirmed this behaviour in both our stocks. In addition, in numerous experiments involving transplantable tumours syngeneic for our BALB/c mice they behaved identically to those from other sources.

Since our mice were undoubtedly BALB/c, the question arises whether they were infected by accidental contact with mouse milk from a colony with virulent MTV or by activation of endogenous virus. In either case the virus was presumably disseminated throughout the colony by 1967.

Bentvelzen *et al.* (1970), Schlom *et al.* (1973) and Pillsbury and Moore (unpublished) have presented evidence of type B particles and MTV in BALB/c. We know of no other published reports of such a high spontaneous incidence of MTV-associated mammary tumours in young BALB/c female breeders. Since BALB/c mice are among the strains most susceptible to MTV and are most frequently used to isolate MTV, our experience should alert other investigators to these possibilities.

### REFERENCES

ANDERVONT, H. B. (1945) Fate of the C3H Milk Influence in Mice of Strains C and C57 Black. *J. natn. Cancer Inst.*, **5**, 383.

ANDERVONT, H. B. (1964) Fate of the C3H Mammary Tumor Agent in Mice of Strains C57BL, I, and BALB/c. *J. natn. Cancer Inst.*, **32**, 1189.

ANDERVONT, H. B. & DUNN, T. B. (1948) Efforts to Detect a Mammary-tumor Agent in Strain C Mice. *J. natn. Cancer Inst.*, **8**, 235.

BENTVELZEN, P., DAAMS, J. H., HAGEMAN, P. & CALAFAT, J. (1970) Genetic Transmission of Viruses that Incite Mammary Tumors in Mice. *Proc. natn. Acad. Sci. U.S.A.*, **67**, 377.

DAWSON, P. J., BROOKS, R. E. & FIELDSTEEL, A. H. (1974) Unusual Occurrence of Endometrial Sarcomas in Hybrid Mice. *J. natn. Cancer Inst.*, **52**, 207.

DERINGER, M. (1965) Occurrence of Mammary Tumors, Reticular Neoplasms and Pulmonary Tumors in Strain BALB/cAnDe Breeding Female Mice. *J. natn. Cancer Inst.*, **35**, 1047.

DUNN, T. B. (1959) Morphology of Mammary Tumors in Mice. In *The Physiopathology of Cancer* 2nd Edn. Ed. F. Homberger. New York: Paul B. Hoeber, Inc. p. 38.

MADISON, R. M., RABSTEIN, L. S. & BRYAN, W. R. (1972) Mortality Rate and Spontaneous Neoplasms in Breeding and Retired Breeder BALB/cCr Mice throughout the Natural Life Span. *Int. J. Cancer*, **10**, 273.

MOORE, D. H., CHARNEY, J. & HOLBEN, J. A. (1974) Titration of Various Mouse Mammary Tumor Viruses in Different Mouse Strains. *J. natn. Cancer Inst.*, **52**, 1757.

NANDI, S. (1963) New Method for Detection of Mouse Mammary Tumor Virus. I. Influence of Foster Nursing on Incidence of Hyperplastic Mammary Nodules in BALB/cCrgl Mice. *J. natn. Cancer Inst.*, **31**, 57.

PETERS, R. L., RABSTEIN, O. S., SPAHN, G. J., MADISON, R. M. & HUEBNER, R. J. (1972) Incidence of Spontaneous Neoplasms in Breeding and Retired Breeder BALB/cCr Mice throughout the Natural Life Span. *Int. J. Cancer*, **10**, 273.

SCHLOM, J., MICHALIDES, R., KUFE, D., HEHLMANN, R., SPIEGELMAN, S., BENTVELZEN, P. & HAGEMAN, P. (1973) A Comparative Study of the Biologic and Molecular Basis of Murine Mammary Carcinoma: a Model for Human Breast Cancer. *J. natn. Cancer Inst.*, **51**, 541.